# Exosomes in oncofertility: emerging roles in chemotherapy-induced reproductive damage and fertility preservation

**DOI:** 10.1530/RAF-25-0149

**Published:** 2026-04-21

**Authors:** Mariam M Abady, Budur Alshehri, Kholoud Aldakhil, Alanoud Alqassim, Islam M Saadeldin

**Affiliations:** ^1^ Center for Gene and Cell Therapy, Korea Research Institute of Bioscience and Biotechnology (KRIBB), Daejeon 34141, Republic of Korea; ^2^ Nutrition and Food Science Department, National Research Centre, Dokki, Giza, Egypt; ^3^ Research Laboratories, King Faisal Specialist Hospital and Research Centre, Riyadh, Saudi Arabia

**Keywords:** oncofertility, exosomes, chemotherapy-induced gonadotoxicity, fertility preservation, reproductive regeneration

## Abstract

**Abstract:**

Oncofertility has emerged as a critical interdisciplinary field addressing the reproductive challenges faced by cancer patients, particularly those undergoing chemotherapy. While chemotherapeutic agents remain indispensable in cancer therapy, their gonadotoxic effects frequently result in diminished ovarian reserve, impaired spermatogenesis, and long-term infertility. Exosomes – small extracellular vesicles enriched with nucleic acids, proteins, and lipids – are increasingly recognized as key mediators of intercellular communication in both pathological and regenerative contexts. Recent evidence suggests that chemotherapy alters exosome cargo, thereby amplifying cellular stress responses, oxidative damage, and bystander effects in gonadal tissues. Conversely, exosomes derived from mesenchymal stem cells, induced pluripotent stem cells (iPSCs), and other regenerative sources demonstrate the ability to restore ovarian and testicular function by reducing apoptosis, enhancing angiogenesis, and supporting germ cell survival. This dual role positions exosomes as both contributors to reproductive toxicity and promising therapeutic agents in fertility preservation strategies. However, clinical translation remains hindered by challenges including source heterogeneity, isolation methods, safety concerns, and regulatory barriers. This review highlights the emerging roles of exosomes in chemotherapy-induced reproductive damage, explores their regenerative potential, and outlines future directions for their integration into oncofertility practice.

**Lay summary:**

Cancer treatments such as chemotherapy save lives, but they often damage the ovaries and testes. This can lead to infertility in both women and men, which is one of the most difficult problems for cancer survivors. Finding ways to protect or restore fertility is, therefore, an important part of cancer care. Our work looks at very small particles called *exosomes*. These are natural messengers that carry proteins and genetic material between cells. We explain how chemotherapy changes these exosomes in a way that increases damage to reproductive organs. At the same time, we show that exosomes from stem cells may help repair this damage. In laboratory studies, they have been shown to protect eggs and sperm and reduce cell death of the reproductive organs during chemotherapy. Exosomes can also act as simple blood-based tests to detect damage and monitor recovery. This research is important because it points to new, less invasive ways to protect fertility during and after cancer treatment. If successful, exosome-based therapies could give patients not only a chance of survival but also the hope of having children in the future.

## Introduction

Advances in cancer therapy have markedly improved survival among patients of reproductive age, bringing long-term quality of life – and fertility preservation – into sharper clinical focus. Oncofertility has emerged as an interdisciplinary field at the interface of oncology and reproductive medicine, aiming to prevent or mitigate treatment-related reproductive damage (La Rosa *et al.* 2020). Despite therapeutic success, chemotherapy remains a major cause of gonadal injury, frequently resulting in reduced ovarian reserve, impaired spermatogenesis, and permanent infertility in cancer survivors.

Several fertility-preserving strategies are currently available, including gonadotropin-releasing hormone agonists, cryopreservation of oocytes or embryos, and ovarian tissue cryopreservation (Zaami *et al.* 2022). While these approaches have expanded reproductive options, they are not universally effective and are often limited by invasiveness, time constraints, patient age, or the urgency of cancer treatment. Consequently, there is a clear need for alternative, less invasive strategies that can protect reproductive function without delaying oncologic care.

**Figure 1:**
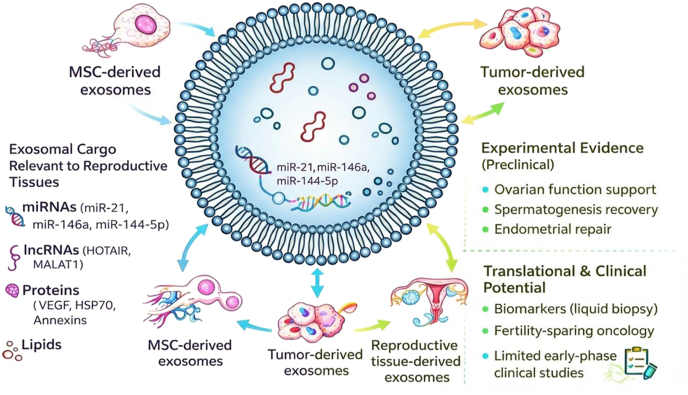
A simplified overview of exosome structure and its diverse molecular cargo, including proteins, lipids, metabolites, and nucleic acids. Through this cargo, exosomes participate in intercellular communication and mirror the physiological or pathological state of their cells of origin. In the context of reproductive biology and oncofertility, exosomes are being explored as non-invasive biomarkers, therapeutic mediators, and delivery vehicles, highlighting their potential role in linking cancer treatment with fertility preservation. The figure was drawn with the Microsoft PowerPoint software, and the quality was enhanced with a scientific illustrator toolkit (FigureLabs*,* https://www.figurelabs.ai).

In this context, exosomes have gained increasing attention as biologically active mediators with relevance to reproductive health. Exosomes are nanosized extracellular vesicles (30–150 nm) of endosomal origin that facilitate intercellular communication through the transfer of proteins, lipids, and regulatory RNAs (Lee *et al.* 2024). They participate in a wide range of physiological and pathological processes, including immune regulation, cancer progression, and reproductive biology (Saadeldin *et al.* 2025) (Fig. 1). Importantly, chemotherapy can alter exosomal cargo, enabling the dissemination of stress signals that may exacerbate gonadal damage beyond direct cellular toxicity.

At the same time, exosomes derived from regenerative sources – such as mesenchymal stem cells (MSCs) and induced pluripotent stem cells – have shown promising protective and reparative effects in experimental models. These vesicles can reduce apoptosis, modulate inflammation, promote angiogenesis, and support germ cell survival, suggesting a potential role in preserving or restoring reproductive function. Together, these findings position exosomes as having a dual role in oncofertility: contributors to chemotherapy-induced reproductive injury and emerging tools for fertility protection (Ludwig & Giebel 2012; Qamar *et al.* 2021).

In this review, we synthesize current knowledge on chemotherapy-induced gonadotoxicity in both females and males, explore the mechanistic and regenerative roles of exosomes in reproductive damage and repair, and discuss the key challenges and future directions for translating exosome-based approaches into oncofertility practice.

## Chemotherapy-induced reproductive damage

Cancer is one of the most pressing global public health challenges, with a rising incidence among younger populations (Siegel *et al.* 2019). For example, between 2001 and 2010, childhood cancers were reported at 140 cases per million individuals under 14 years (Steliarova-Foucher *et al.* 2017). Advances in screening and treatment have markedly improved survival, with an ∼20% increase over the past three decades (Xiao *et al.* 2017).

However, the expanding population of survivors faces long-term adverse effects of chemotherapy, including neurocognitive impairment, cardiotoxicity, secondary malignancies, and notably, gonadotoxicity, which threatens fertility, endocrine function, and quality of life (Silva *et al.* 2018). Chemotherapy-induced reproductive toxicity arises through mechanisms such as DNA damage, oxidative stress, germ cell apoptosis, and disruption of the gonadal microenvironment. In females, this manifests as loss of the ovarian reserve and premature ovarian insufficiency, whereas in males it leads to impaired spermatogenesis, poor sperm quality, or azoospermia.

The extent of gonadotoxicity varies by drug class, cumulative dose, treatment duration, and age at exposure, with alkylating agents recognized as the most damaging. Common regimens include single agents, such as cyclophosphamide, doxorubicin, paclitaxel, and cisplatin, or combinations, such as MOPP and ABVD for Hodgkin’s lymphoma and CHOP for non-Hodgkin’s lymphoma (Hao *et al.* 2019). While effective in cancer control, these regimens inevitably compromise reproductive health, underscoring the need for fertility-preserving interventions.

Having outlined the general mechanisms and regimens, the following section examines the drug-specific gonadotoxic profiles, highlighting how individual agents exert distinct reproductive effects based on their molecular targets.

### Impact of chemotherapeutic agents on female fertility

Doxorubicin, an anthracycline antibiotic derived from *Streptomyces peucetius* and marketed as Adriamycin or Rubex, is a widely used chemotherapeutic for leukemia, breast, lung, stomach, and ovarian cancers (Spears *et al.* 2019). Its anti-tumor activity stems from DNA intercalation, topoisomerase II inhibition, and disruption of mitochondrial electron transport, leading to free radical generation and oxidative stress (Bhardwaj *et al.* 2020). However, doxorubicin is profoundly ovotoxic, causing amenorrhea, premature ovarian failure (POF), and infertility in female cancer survivors (Molina *et al.* 2005). Animal studies demonstrate dose-dependent reductions in ovarian and uterine weight, follicular atresia, peri-ovarian edema, and impaired ovulation, largely linked to oxidative stress (Nishi *et al.* 2018). Histology reveals stromal and vascular damage, cortical fibrosis, and uterine abnormalities (Bhardwaj *et al.* 2023). Doxorubicin also disrupts estrous cycles, lowers estrogen levels, and impairs mammary gland development (Nishi *et al.* 2018). Mechanistically, it crosses the blood–follicle barrier, accumulates in granulosa cells and oocytes, and induces mitochondrial dysfunction, DNA double-strand breaks, ER stress, and Ca^2+^ overload, activating caspase-3, caspase-12, and cytochrome c-mediated apoptosis (Aziz *et al.* 2019). Moreover, doxorubicin alters steroidogenesis by dysregulating FOXO3/AKT signaling, modifying miRNA expression (e.g. miR-132-3p), and downregulating key enzymes such as P450scc and aromatase, reducing progesterone and estrogen synthesis (Wang *et al.* 2019*b*, Al-Kawlani *et al.* 2020). Deficiency of MDR1, normally expressed in ovarian and uterine tissue, further exacerbates its toxicity (Duan *et al.* 2012). Collectively, these findings establish doxorubicin as a major contributor to female reproductive failure through oxidative stress, DNA damage, ER stress, apoptosis, and steroidogenic disruption.

Cyclophosphamide, an alkylating agent used for lymphomas, breast cancer, and autoimmune disorders, was the first drug linked to amenorrhea and ovarian failure (Li *et al.* 2019*c*). As a prodrug, it is metabolized by hepatic cytochrome P-450 into phosphoramide mustard (PM) and acrolein – potent ovotoxins. PM intercalates into DNA, causing crosslinks and extensive ovarian damage, while both PM and chloroethylaziridine accelerate primordial and primary follicle loss (Ponticelli *et al.* 2018). Animal and clinical studies show reduced ovarian/uterine weight, granulosa cell arrest, and nearly tenfold increased risk of ovarian insufficiency. Histology reveals stromal edema, vascular injury, fibrosis, and follicular atresia (Ganesan & Keating 2015, Abdel-Aziz *et al.* 2020). Mechanisms include oxidative stress, DNA double-strand breaks, apoptosis, and inflammatory cytokines (TNF-α, IL-1, and IL-6), with caspase-3 activation and altered antioxidant enzymes (SOD, GSH, and HO-1) promoting apoptosis. Endocrine disruption – reduced estradiol, progesterone, FSHR, and PCNA – further impairs folliculogenesis and embryo development (Delkhosh *et al.* 2019, Bhardwaj *et al.* 2023). Thus, cyclophosphamide induces severe gonadotoxicity via DNA crosslinking, oxidative/inflammatory stress, and hormonal imbalance, culminating in POF and infertility.

Cisplatin, a platinum-based chemotherapeutic approved in 1978, is widely used for ovarian, breast, lung, bladder, and testicular cancers (Bhardwaj *et al.* 2023). Its cytotoxicity arises from DNA crosslinking at guanine/adenine residues, blocking replication, impairing repair, and inducing apoptosis. Despite its efficacy, cisplatin causes severe female reproductive toxicity, including embryonic lethality, blastocyst defects, teratogenicity, and growth retardation (Hassan *et al.* 2019). Mechanistically, cisplatin promotes granulosa cell apoptosis, depletes primordial follicles, and leads to follicular degeneration, vascular congestion, and oocyte loss (Kulhan *et al.* 2019). It also drives oxidative stress – reducing superoxide dismutase and glutathione while elevating lipid peroxidation and reactive oxygen species (ROS) (Bhardwaj *et al.* 2023). Endocrine disruption occurs via PTEN/Akt/FOXO3a signaling, accelerating premature follicle activation and reducing AMH and inhibin levels. In parallel, apoptotic pathways involving p53, ATM, and MAPK signaling activate TAp63α in oocytes, upregulating PUMA and NOXA and triggering follicular apoptosis (Jang *et al.* 2017, Bhagat *et al.* 2021). Together, these findings confirm cisplatin-induced infertility results from a combination of oxidative stress, endocrine disruption, and apoptosis, ultimately causing irreversible ovarian reserve loss.

Paclitaxel, a natural chemotherapeutic from *Taxus brevifolia*, is widely used for breast, ovarian, lung, and germ cell tumors (Ettinger *et al.* 2019). Unlike alkylating or platinum agents, it stabilizes microtubules and prevents depolymerization, blocking cell division but rendering granulosa cells highly vulnerable (Zhang *et al.* 2017). Paclitaxel reduces primordial follicle counts, induces atresia, and causes ovarian failure (Kim *et al.* 2019). It suppresses GDF9 and BMP15 transcription, disrupts granulosa cells, and triggers lipid accumulation and mitochondrial injury. Antral follicles and MII oocytes are especially sensitive, showing meiotic arrest, spindle abnormalities, and chromosomal misalignment, leading to reduced fertilization and pregnancy rates (Ma *et al.* 2020). At the molecular level, paclitaxel inhibits CDK1 and cyclin A, causing G2/M arrest, and promotes apoptosis via Bcl-2 and XIAP downregulation alongside pro-apoptotic gene activation. It also elevates PARP cleavage and DNA damage markers, further impairing oocyte quality *et al.*, 2019). Endocrine effects include reduced estrogen with relatively stable progesterone levels (Palomino *et al.* 2024). Overall, paclitaxel impairs female fertility by stabilizing microtubules, inducing follicular loss, triggering apoptosis, and disrupting hormonal balance.

### Impact of chemotherapeutic agents on male fertility

Although much of the clinical literature emphasizes female reproductive damage, males are also significantly affected by chemotherapy-induced gonadotoxicity. Male fertility experiences two main chemotherapy side effects which result in oligospermia and azoospermia leading to possible long-term or short-term infertility. The two alkylating agents, cyclophosphamide (CP) and busulfan (BF), serve as primary treatments for pediatric cancer patients who have multiple myeloma and sarcomas, chronic myeloid leukemia, and lymphomas. Male cancer survivors who become infertile experience psychological damage that affects their self-esteem and emotional health, their relationships, social position, financial security, and life quality (Liakath Ali *et al.* 2023).

The causes of male infertility associated with cancer treatment can be broadly categorized into four groups: i) testicular dysfunction, where the testes fail to produce adequate sperm; ii) obstruction or structural abnormalities of the seminal tract; iii) sexual dysfunction, including erectile dysfunction and ejaculatory disorders (Yumura *et al.* 2018); and iv) hypogonadism, resulting from reduced gonadotropin secretion and impaired spermatogenesis, which may arise after cranial radiation therapy, central nervous system tumors, or surgical interventions, such as partial or total orchiectomy (Elenkov & Giwercman 2022). Moreover, hypogonadism can also be triggered by pituitary inflammation caused by immune checkpoint inhibitors, such as nivolumab (Özdemir 2021, Yumura *et al.* 2023).

Testicular dysfunction remains one of the most critical long-term complications of chemotherapy and radiotherapy in young male cancer survivors. Schrader *et al.* (2001) reported that 15–30% of survivors lose fertility potential, with many showing abnormal semen parameters. For example, oligozoospermia has been observed in 28% of testicular cancer, 25% of Hodgkin’s disease, 57% of leukemia, and 33% of gastrointestinal cancer patients (Chung *et al.* 2004). Among those undergoing sperm banking, about 50% have sperm counts ≤10 million (Williams *et al.* 2009), while 5–11% present with azoospermia (Johnson *et al.* 2013). Poor nutrition, endocrine abnormalities, and elevated cytokine levels may further exacerbate sperm decline, and these conditions often persist even after treatment (Dohle 2010, Yumura *et al.* 2023).

Studies indicate that chemotherapeutic agents such as cyclophosphamide, cisplatin, and doxorubicin can induce apoptosis in spermatogonial stem cells (SSCs) by causing DNA damage (Smart *et al.* 2018). In particular, drugs such as cyclophosphamide, cisplatin, etoposide, and vincristine predominantly activate programmed cell death in spermatogonia and primary spermatocytes (Park *et al.* 2022). Doxorubicin, however, may also elicit testicular injury through alternative mechanisms, including necrosis or autophagy (Allen *et al.* 2018, Ghafouri-Fard *et al.* 2021). While Sertoli cells can be directly affected, their dysfunction often arises secondarily due to germ cell loss. Beyond direct cytotoxicity, chemotherapy also provokes cellular stress within the testes, leading to impaired testicular function and activation of apoptotic signaling cascades. These effects are further amplified by enhanced pro-inflammatory cytokine release, nuclear translocation of NF-κB, and suppression of the antioxidant regulator Nrf2, ultimately promoting oxidative stress and gonadal damage (Ghafouri-Fard *et al.* 2021, Taher *et al.* 2025).

Surgical interventions can also impair fertility: prostatectomy or cystectomy may cause aspermia, retroperitoneal lymph node dissection can lead to anejaculation, radical prostatectomy often results in erectile dysfunction, and bilateral orchiectomy causes irreversible azoospermia (Huang & Berg 2021). Oxidative stress is another contributor, with reactive oxygen species detected in semen of 42% of chemotherapy-treated patients, persisting long after therapy (Takeshima *et al.* 2019, Yumura *et al.* 2023).

Large-scale studies confirm the elevated risk: in a cohort of 1,622 survivors, infertility prevalence was 46% compared with 17.5% in siblings (RR = 2.34) (Wasilewski-Masker *et al.* 2014), aligning with the general infertility rate of ∼15% in developed countries (Sharlip *et al.* 2002, Yumura *et al.* 2023). Notably, infertility in survivors was largely linked to gonadotoxic therapies rather than baseline causes, with additional risks from genital or spinal surgeries (Wasilewski-Masker *et al.* 2014). Chemotherapy exposure in both childhood and adulthood has been associated with semen deterioration, increased sperm DNA fragmentation, and reduced birth rates (Delessard *et al.* 2020). While cancer treatment rightly prioritizes survival, these findings underscore the need to anticipate and mitigate infertility risks in male patients.

Spermatogenesis in seminiferous tubules depends on Sertoli cell support and the blood–testis barrier, but many drugs – especially alkylating agents, such as ethyl methanesulfonate, and tyrosine kinase inhibitors, such as imatinib – can cross and damage germ cells (Chang *et al.* 2021). Differentiating spermatogonia are highly sensitive, while late-stage germ cells are more resistant (Trottmann *et al.* 2007, Yumura *et al.* 2023). Sperm counts can drop to 1/10–1/100 of normal within 1–2 months of therapy, with azoospermia appearing in ∼12 weeks depending on drug and dose (Meistrich 2013, Yumura *et al.* 2023). Recovery is regimen-dependent: mild treatments may normalize counts in ∼12 weeks, but alkylating agents often cause lasting or irreversible damage (Meistrich 2013, Mulder *et al.* 2021). Cumulative dose strongly predicts toxicity – CED ≥ 4,000 mg/m^2^ links to azoospermia; ifosfamide > 42 g/m^2^ and cisplatin >400 mg/m^2^ are also highly gonadotoxic (Green *et al.* 2014). Targeted therapies may impair fertility too, since sperm rely on tyrosine kinases for meiosis and capacitation, though data remain limited (Wyns *et al.* 2021).

While the mechanisms and clinical outcomes of chemotherapy-induced reproductive toxicity are well documented, mounting evidence suggests that these effects extend beyond direct cellular injury. Extracellular vesicles, especially exosomes, have emerged as key mediators that amplify gonadotoxic signals. By transferring proteins, lipids, and regulatory RNAs between cells, exosomes act as intercellular messengers that shape the onset, progression, and persistence of chemotherapy-induced damage. This underscores the need to explore in depth the role of exosomes in mediating chemotherapy-induced reproductive toxicity.

## Role of exosomes in chemotherapy-induced reproductive toxicity

### Origin and biogenesis of exosomes

Exosomes were first described in the early 1980s in reticulocyte culture media and are now recognized as nanoscale vesicles present in nearly all body fluids. According to MISEV 2023, small extracellular vesicles (sEVs) include exosomes (<200 nm), derived from the endosomal system, and ectosomes, which bud directly from the plasma membrane and often overlap in size (Taher *et al.* 2025).

Exosome formation begins with endosomal trafficking, where inward budding of early endosomes generates intraluminal vesicles (ILVs) that accumulate within multivesicular bodies (MVBs). These MVBs fuse with either lysosomes for degradation or the plasma membrane to release ILVs as exosomes (Gurung *et al.* 2021). Their biogenesis is largely governed by the endosomal sorting complex required for transport (ESCRT), comprising ESCRT-0 to ESCRT-III, together with accessory proteins such as VPS4, TSG101, and ALIX (Gurung *et al.* 2021). ESCRT-0 captures ubiquitinated cargos, ESCRT-I/II induce membrane deformation, and ESCRT-III with VPS4 mediates scission. ALIX and TSG101 also contribute to selective cargo loading by interacting with syndecans (Hessvik & Llorente 2018, Yue *et al.* 2020).

Alongside this canonical pathway, ESCRT-independent mechanisms have been identified. Lipid-driven processes involving sphingomyelinase-derived ceramides promote membrane curvature and budding (Skryabin *et al.* 2020), while tetraspanins such as CD63 and CD81 cluster receptors and signaling proteins in microdomains, guiding their incorporation into exosomes (Mohammadi *et al.* 2024). These dual pathways highlight the versatility of exosome biogenesis, with the balance between ESCRT-dependent and lipid/tetraspanin-driven processes varying by cell type and physiological context (Mohammadi *et al.* 2024).

Unlike exosomes, ectosomes form by direct budding from the plasma membrane, involving lipid-anchored proteins (e.g., myristoylation/palmitoylation), cargo clustering, and ESCRT-I recruitment (Lim *et al.* 2021). Key regulators include small GTPases such as Arf6 (vesicular trafficking) and Rho family proteins (RhoA, Rac1, and Cdc42) that remodel cortical actin (Meldolesi 2018). Final scission requires Ca^2+^-dependent lipid rearrangements (via flippases) and ESCRT-III activity (Yu *et al.* 2024).

### Molecular cargo of exosomes

sEVs carry a molecular profile reflecting their parent cells, including proteins, lipids, and nucleic acids (Rahimian *et al.* 2024). Databases such as EVpedia, Vesiclepedia, and ExoCarta catalog their contents.

#### Lipid content

sEV membranes are enriched in sphingomyelin, gangliosides, phosphatidylserine, and ceramide but show reduced phosphatidylcholine and diacylglycerol. These features, along with cholesterol enrichment, confer greater rigidity and stability compared to the cell membrane (Chen & Yu 2022). Exosomes are enriched in tetraspanins and ICAM-1, while ectosomes carry a wider array of proteins, including glycoproteins and metalloproteinases (Taher *et al.* 2025).

#### Protein content

sEV proteins reflect their biogenesis, often including ESCRT components, tetraspanins, and transmembrane signaling molecules. Proteins from the nucleus, Golgi, and ER are generally absent (Abels & Breakefield 2016).

#### Nucleic acid content

sEVs transport diverse RNAs, including mRNA, miRNA, lncRNA, tRNA, circRNA, and snRNA, as well as double-stranded DNA (Chen *et al.* 2021*b*). These molecules regulate gene expression and are promising biomarkers (Taher *et al.* 2025).

Their distinct size, cargo, and biogenesis differentiate them from microvesicles and apoptotic bodies (Aheget *et al.* 2021). Morphologically, they appear cup-shaped under preparation but spherical in TEM (Yellon & Davidson 2014). Their density ranges from 1.13 g/mL (B-cell exosomes) to 1.19 g/mL (epithelial-cell exosomes) (Zakharova *et al.* 2007).

Exosomes are nanosized vesicles that mediate the transfer of molecular cargos – such as proteins, lipids, and nucleic acids – from donor cells to recipient cells, thereby serving as a central mechanism of intercellular communication (Hannafon & Ding 2013, Shirani *et al.* 2025). A wide variety of cells, including dendritic cells, macrophages, cancer cells, and MSCs, exploit this pathway to regulate both local and systemic signaling. Once secreted, exosomes profoundly influence cellular functions, as their cargos can induce new biological responses in recipient cells, thereby modulating key mechanisms in both health and disease (Gurung *et al.* 2021).

Following release into the extracellular matrix (ECM), exosomes reach target cells via multiple routes, including autocrine, juxtacrine, paracrine, or endocrine signaling (Hao *et al.* 2022). Uptake mechanisms depend on the recipient cell type and typically occur through two major modes. In the first, exosomes bind directly to surface receptors (e.g., glycans, lectins, integrins, and adhesion molecules), thereby activating downstream signaling pathways without internalization. In the second, internalization occurs through various forms of endocytosis, including clathrin-dependent or clathrin-independent endocytosis, phagocytosis, and macropinocytosis. Once internalized, exosomes may be degraded in lysosomes, recycled back to the plasma membrane, or release their cargos into the cytoplasm, thereby influencing recipient cell function (Gurung *et al.* 2021, Shirani *et al.* 2025).

### Exosome isolation and characterization

The isolation of exosomes is a critical step for obtaining pure and concentrated vesicles for research (Ayala-Mar *et al.* 2019). Several techniques are commonly employed. Differential ultracentrifugation remains the gold standard, separating vesicles by size and density (Huang *et al.* 2022). Ultrafiltration, using molecular weight cutoff membranes, offers faster processing of large volumes (Diaz *et al.* 2018, Abady *et al.* 2023). Size exclusion chromatography separates exosomes through porous beads, while immunocapture methods use antibodies against exosomal markers, providing high specificity but limited by antibody availability (Logozzi *et al.* 2020, Abady *et al.* 2025). More recently, microfluidic platforms have emerged, allowing size- and marker-based isolation with rapid processing and integration into downstream analyses (Kumar *et al.* 2023). No single method ensures complete purity; thus, combining methods or using commercial kits often improves results (Ayala-Mar *et al.* 2019, Mohammadi *et al.* 2024).

To assess purity and efficiency, density gradient centrifugation is widely used to separate exosome subpopulations (Willms *et al.* 2016). Exosomal markers such as tetraspanins (CD9, CD63, and CD81) help confirm identity and purity (Schorey *et al.* 2015). Molecular profiling of RNA and miRNAs provides additional insights, distinguishing healthy from diseased states (Mohammadi *et al.* 2024). Morphology and protein cargo can be validated using TEM and Western blotting (Jiao *et al.* 2019). Lipid profiling, including cholesterol and sphingolipids, further contributes to quality assessment (Yasuda *et al.* 2022).

### Exosomes in infertility pathogenesis and diagnosis

Exosomes are present in nearly all biological fluids, and their contents can be analyzed through liquid biopsies (Jahanbani *et al.* 2023*b*). Their molecular cargo – including miRNAs, proteins, and lipids – serves as a unique ‘fingerprint’ of the donor cell, reflecting both its cellular origin and physiological state (Yu *et al.* 2021). Because of these features, exosomes are increasingly recognized as non-invasive diagnostic and prognostic biomarkers (Yu *et al.* 2022). They hold great promise for the detection of diseases affecting the reproductive system, liver, cardiovascular system, and various cancers (Pillay *et al.* 2017).

**Figure 2:**
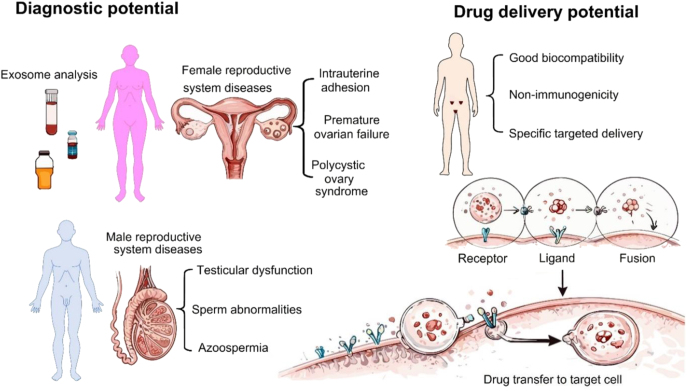
Diagnostic and therapeutic roles of exosomes in reproductive disorders. The left panel shows the use of exosome analysis as a non-invasive diagnostic tool for female and male reproductive disorders, including intrauterine adhesion, POF, polycystic ovary syndrome, testicular dysfunction, sperm abnormalities, and azoospermia. The right panel highlights the therapeutic potential of exosomes as drug delivery systems to the reproductive organs, characterized by biocompatibility, low immunogenicity, and targeted delivery of therapeutic cargo to recipient cells. The figure was drawn with the Microsoft PowerPoint software, and the quality was enhanced with a scientific illustrator toolkit (FigureLabs, https://www.figurelabs.ai).

Exosomes are increasingly recognized as central regulators in female reproductive disorders through their ability to transfer RNAs, proteins, and other cargos that alter cell behavior (Fig. 2). In reproductive medicine, exosomes have demonstrated diagnostic potential for conditions such as polycystic ovary syndrome (PCOS), POF, endometriosis, intrauterine adhesion (IUA), Asherman’s syndrome, and preeclampsia (Vickram *et al.* 2021, Yu *et al.* 2022). For example, exosomal RNA sequencing from human follicular fluid (HFF) has shown potential as a molecular biomarker for PCOS (Hu *et al.* 2020). Similarly, a serum exosome profiling study revealed 54 miRNAs with significant differences between PCOS patients and controls, introducing hsa-miR-1299, hsa-miR-6818-5p, hsa-miR-192-5p, and hsa-miR-145-5p as potential biomarkers (Zhang *et al.* 2021*b*).

In PCOS, which affects 6–8% of women (Esfandyari 2020), exosomal miRNAs such as miR-25-3p and miR-143-3p are upregulated while others such as miR-10a-5p are downregulated, thereby reshaping metabolic pathways (Hu *et al.* 2020). Likewise, serum exosomal miR-146a-5p and miR-126-3p influence MAPK and circadian signaling (Jiang *et al.* 2021), whereas adipose exosomal miR-323-3p protects granulosa cells by targeting PDCD4 (Zhao *et al.* 2019). Notably, circRNAs such as hsa-circ-0006877 and proteins such as S100-A9 further implicate exosomes in inflammation and insulin resistance (Tehrani *et al.* 2021).

Moving to POF, which occurs in 1% of women between 30 and 39 years (Zhang 2020), MSC-derived exosomes demonstrate restorative potential. For example, bone marrow MSC exosomal miR-144-5p regulates PTEN (Sun *et al.* 2019), hAEC-derived miR-1246 activates PI3K/AKT (Zhang *et al.* 2019*b*), and amniotic fluid stem cell (AFSC)-derived exosomes transfer miR-10a and miR-146a to inhibit apoptosis. Placental MSC exosomes also enhance antioxidant defenses such as catalase and PRDX1, offering further protection (Seok *et al.* 2020).

Importantly, recent mechanistic studies have identified AFSC-derived extracellular vesicles enriched in miR-21 as potent mediators of ovarian regeneration in chemotherapy-induced POF models. These EVs restored follicular counts, normalized serum anti-Müllerian hormone (AMH) levels, and improved fertility outcomes, primarily through suppression of the PTEN/caspase-3 apoptotic pathway. Notably, selective loading of AFSC-EVs with miR-21 mimics reproduced the regenerative effects, whereas inhibition of miR-21 abrogated ovarian recovery, underscoring the functional relevance of exosomal miRNA cargo in mediating therapeutic efficacy (Thabet *et al.* 2020).

Beyond therapy, exosomes also hold promise in POF diagnosis. Reduced levels of Yy2 mRNA in peripheral blood-derived exosomes have been reported in POF patients, with expression levels correlating with disease severity and hormonal status (Liu *et al.* 2021). In IUA, miR-326 has emerged as both a prognostic biomarker and a therapeutic target, as its downregulation correlates with enhanced fibrosis, while its overexpression suppresses the TGF-β1/Smad3 signaling pathway (Javadi *et al.* 2022). Although no specific exosome biomarker has yet been confirmed for IUA, miR-326 remains a promising candidate.

Exosomes also show potential in endometriosis research, a condition lacking reliable diagnostic biomarkers. Investigations into miRNA signatures have been promising, with studies identifying five unique miRNAs in endometriotic epithelial cells, absent in healthy tissues (Shomali *et al.* 2020). This supports exosome analysis as a tool for understanding endometriosis pathophysiology and improving diagnosis.

In Asherman’s syndrome, MSC-derived exosomes reduce fibrosis and stimulate angiogenesis by modulating MMP-2/9, VEGFR1, and CD31 while suppressing TIMP-2, suggesting a novel therapeutic approach (Saribas *et al.* 2020). In endometriosis, affecting 6–10% of reproductive-aged women, exosomal lncRNAs (aHIF and CHL1-AS1) and miRNAs (miR-22-3p and miR-214-3p) regulate angiogenesis and fibrosis (Zhang *et al.* 2021*a*), while proteins such as CD47 and PRDX1 show diagnostic potential (Nazri *et al.* 2020). Macrophage- and stromal-derived exosomes further promote immune evasion and lesion progression (Zhang *et al.* 2021*a*). In endometrial cancer, tumor and stromal exosomes transfer oncogenic cargos: CAF exosomes suppress miR-148b/miR-320a, activating DNMT1 and VEGFA (Zhang *et al.* 2020*a*), plasma exosomal LGALS3BP stimulates PI3K/AKT/VEGFA signaling (Song *et al.* 2021), and hypoxic exosomes enriched in miR-21 polarize macrophages (Xiao *et al.* 2020). Urinary exosomal miR-200c-3p and circ_0109046 are promising non-invasive biomarkers (Shi *et al.* 2020).

In cervical cancer, HPV-driven exosomes carry survivin (Honegger *et al.* 2015) and miRNAs (miR-21, miR-146a, and miR-221) that regulate EMT, angiogenesis, and metastasis. lncRNAs such as HOTAIR and MALAT1 (Guo *et al.* 2020) and proteins linked to Hedgehog and RAS signaling (Bhat *et al.* 2018) enhance drug resistance and progression. In ovarian cancer, the deadliest gynecological malignancy, exosomes are enriched in CD9, CD63, HSPs, EpCAM, and CA-125 (Wyciszkiewicz *et al.* 2019). They drive angiogenesis, metastasis, and platinum resistance via annexin A3 (Yin *et al.* 2012). Exosomal miRNAs (miR-21, miR-200, and miR-221) regulate invasion, drug resistance, and angiogenesis, with several (e.g., miR-200f and miR-21) being tested as liquid biopsy markers (Yoshida *et al.* 2020).

In preeclampsia, responsible for 10–15% of fetal deaths, placental exosomes are elevated (Salomon & Rice 2017). They carry altered syncytin proteins (Pillay *et al.* 2016), pro-coagulant tissue factors (Gardiner *et al.* 2011), and dysregulated miRNAs (↓miR-23a-3p, ↓miR-144-3p, ↑let-7a-5p, and ↑miR-221-3p) that impair angiogenesis and trophoblast invasion (Truong *et al.* 2017). Conversely, MSC-derived exosomes enriched in VEGF or miR-18b promote vascular repair (Esfandyari *et al.* 2021). Placental trophoblast-derived exosomes containing disease-specific miRNAs (hsa-miR-525-5p, hsa-miR-526b-5p, and hsa-miR-1269b) are being explored as early biomarkers (Truong *et al.* 2017, Jahanbani *et al.* 2023*b*).

The diagnosis of male infertility-related disorders has shown encouraging preliminary outcomes through exosome research. Several studies highlight that exosome-associated proteins, particularly annexin II, play a critical role in male fertility and may serve as promising biomarkers for male infertility disorders (Kowalczyk *et al.* 2022). Early evidence from Diamandis *et al.* (1999) demonstrated that levels of prostaglandin D2 synthase (PTGDS), an enzyme-coding gene product, declined progressively from healthy individuals to azoospermic patients, reaching almost undetectable levels in vasectomized men (Diamandis *et al.* 1999). Follow-up studies further confirmed the diagnostic potential of PTGDS, showing that its expression was significantly reduced in obstructive azoospermia (OA) patients compared to those with non-obstructive azoospermia (NOA) (Heshmat *et al.* 2008) (Fig. 2).

Overall, research investigating the role of exosomes in the pathophysiology of reproductive system disorders is advancing rapidly and has yielded valuable insights. However, despite these promising findings, the clinical application of exosomes as FDA-approved biomarkers remains unrealized, emphasizing the need for further validation through large-scale and standardized studies (Jahanbani *et al.* 2023*a*).

### Sex-dependent reproductive effects of chemotherapy-associated exosomes

Exosomes are abundant in circulating body fluids and reflect the molecular state of their cells of origin, carrying diverse cargos such as proteins, RNAs, and DNA (Feng *et al.* 2019). Beyond their established role as cancer biomarkers, exosomes are now recognized as key mediators of intercellular communication, influencing immune regulation, cellular survival, and tissue homeostasis (Alharbi *et al.* 2021). In the context of chemotherapy, circulating exosomes have emerged as important contributors to systemic, sex-specific reproductive outcomes by transmitting bioactive signals that affect gonadal function and fertility-related processes.

Experimental evidence indicates that exosomes derived from human umbilical cord MSCs (h-UCMSC-Exo) can protect testicular tissue from chemotherapy-associated injury. Using *in vitro* Sertoli cell models and prepubertal mouse models exposed to cyclophosphamide and busulfan, h-UCMSC-Exo preserved the SSC niche, reduced cellular damage, and improved fertility outcomes. Repeated exosome administration was associated with enhanced testosterone production and higher fertility success rates, highlighting a potential fertility-preserving strategy for prepubertal boys undergoing chemotherapy (Liakath Ali *et al.* 2023). Given the sex-dependent nature of reproductive vulnerability and recovery, Table 1 summarizes the major exosome-mediated effects of chemotherapy on female and male reproductive systems.

**Table 1:** Sex-specific reproductive effects of chemotherapy-associated exosomes.

Aspect	Female reproductive system	Male reproductive system	References
Primary targets	Ovarian follicles, granulosa cells, endometrium	Spermatogonial stem cells, Sertoli and Leydig cells	He *et al.* (2019*a*), Liakath Ali *et al.* (2023)
Immune modulation	TAM polarization, T-cell regulation, cytokine balance	Testicular immune niche, inflammatory signaling	Mashouri *et al.* (2019), Masoumi-Dehghi *et al.* (2020)
Hormonal effects	Indirect effects on ovarian reserve and tissue remodeling	Testosterone recovery and endocrine support	Liakath Ali *et al.* (2023), Liang *et al.* (2021)
Functional outcome	Tissue resilience and recovery after chemotherapy	Preservation of spermatogenesis and fertility	Feng *et al.* (2019), Zhang *et al.* (2019*b*)

#### Female reproductive system

In females, chemotherapy-associated exosomes have been extensively studied in reproductive system cancers, where they also provide insight into systemic reproductive effects relevant to oncofertility. Exosomal proteins and non-coding RNAs participate in immune modulation, cellular survival, and tissue remodeling within the ovarian and uterine microenvironments.

Proteomic profiling of exosomes from female reproductive cancers has identified cargos linked to angiogenesis, immune regulation, and cellular stress responses, processes that are closely related to ovarian and endometrial function. Similarly, exosomal miRNAs regulate signaling pathways involved in cell survival, inflammation, and vascular remodeling, thereby shaping tissue responses following chemotherapy exposure (He *et al.* 2019*b*). Beyond miRNAs, exosomal lncRNAs and circRNAs contribute to transcriptional and epigenetic regulation, influencing apoptosis, DNA repair, and immune responses, all of which are critical determinants of reproductive tissue resilience after systemic therapy (Alharbi *et al.* 2021).

Importantly, immune-modulatory exosomes derived from tumor and stromal cells influence macrophage polarization, T-cell activity, and cytokine secretion in ovarian and uterine tissues. These immune effects may indirectly affect follicular survival, endometrial receptivity, and long-term reproductive outcomes following chemotherapy (Masoumi-Dehghi *et al.* 2020). Collectively, these findings suggest that chemotherapy-associated exosomes contribute to female reproductive vulnerability or recovery through coordinated effects on immune balance, cellular stress responses, and tissue remodeling.

#### Male reproductive system

In males, semen represents a unique source of reproductive exosomes, with a substantial proportion originating from the prostate and accessory glands (Roberts *et al.* 2011, Barceló *et al.* 2019). Seminal exosomes carry proteins and regulatory RNAs that influence spermatogenesis, sperm maturation, and immune regulation within the male reproductive tract.

Chemotherapy-associated alterations in exosomal cargo have been linked to changes in germ cell survival, endocrine signaling, and testicular immune homeostasis. Exosomal miRNAs regulate apoptosis, proliferation, and oxidative stress responses in spermatogenic and somatic testicular cells, processes that are central to fertility preservation following gonadotoxic treatments (Mashouri *et al.* 2019). In addition, exosome-mediated communication between Sertoli and Leydig cells influences testosterone synthesis and testicular recovery after systemic chemotherapy exposure (Liang *et al.* 2021).

Prostate-derived exosomes (prostasomes) further illustrate how circulating and seminal exosomes reflect systemic treatment effects while directly modulating male reproductive function. Although extensively studied in prostate cancer, their ability to regulate immune evasion, lipid metabolism, and cellular stress responses also has implications for reproductive health in cancer survivors (Ronquist & Nilsson 2004, Barceló *et al.* 2019).

## Exosomes in regenerative and therapeutic strategies in oncofertility

**Figure 3:**
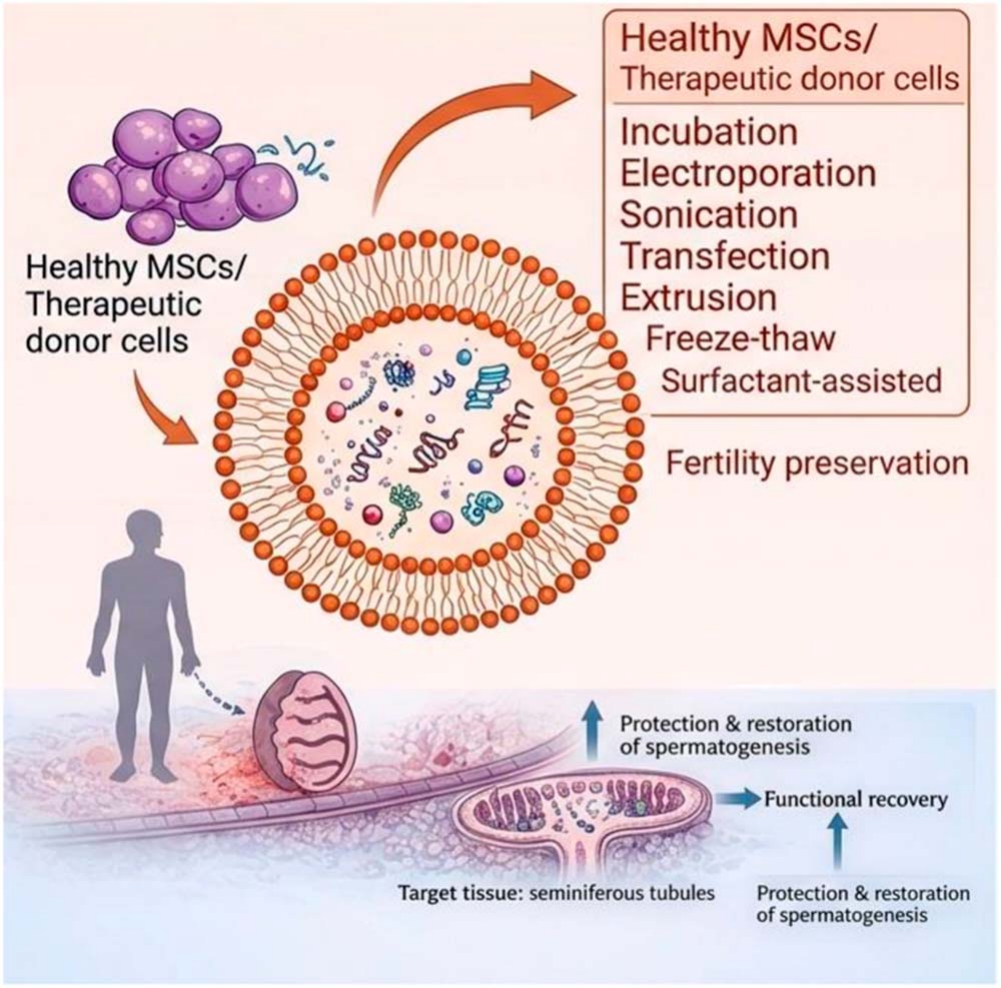
Therapeutic roles of exosomes in oncofertility. Engineered exosomes from healthy mesenchymal stem cells can be loaded with therapeutic molecules (middle) and delivered to chemotherapy-damaged testes to support germ cell survival, restore spermatogenesis, and preserve fertility (right). The figure was drawn with the Microsoft PowerPoint software, and the quality was enhanced with a scientific illustrator toolkit (FigureLabs*,* https://www.figurelabs.ai).

Exosomes are increasingly recognized as active mediators of tissue repair and functional recovery, making them attractive tools for fertility preservation and restoration. As naturally occurring nanovesicles secreted by many cell types, exosomes deliver proteins, lipids, and regulatory RNAs that can promote cell survival, modulate inflammation, enhance angiogenesis, and support tissue remodeling (Kalluri & LeBleu 2020). Compared with stem cell transplantation, exosome-based therapy offers a cell-free and more controllable approach with reduced concerns related to immune rejection and uncontrolled cell behavior (Haider & Aramini 2020, Haider & Aramini 2020, Kalluri & LeBleu 2020). In reproductive medicine, exosomes derived from reproductive tissues (e.g. endometrium, follicular cells, embryos, oviduct, and seminal plasma) contribute to physiological communication (Sun *et al.* 2019); however, for therapeutic applications, MSC-derived exosomes (from bone marrow, adipose tissue, umbilical cord, placenta, and amniotic sources) are most widely investigated because they are accessible and show strong regenerative activity (Fig. 3) (Chen & Yu 2022) (Table 2).

**Figure 4:**
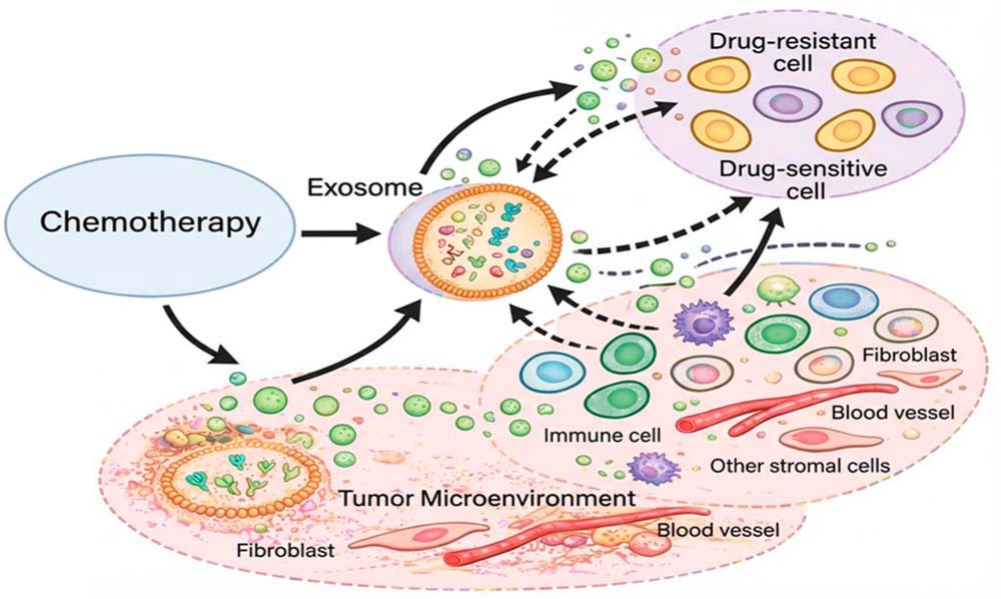
Exosome-mediated crosstalk in the tumor microenvironment during chemotherapy. Exosomes released from chemotherapy-stressed tumor cells interact with drug-sensitive and drug-resistant cells, as well as surrounding stromal cells, transferring bioactive cargo that reshapes the microenvironment and promotes survival and drug resistance. The figure was drawn with the Microsoft PowerPoint software, and the quality was enhanced with a scientific illustrator toolkit (FigureLabs, https://www.figurelabs.ai).

**Table 2:** Overview of exosomal cargos (proteins, miRNAs, lncRNAs, circRNAs, and DNAs) and their functions in female reproductive system cancers.

Cancer	Cargo type	Molecule/marker	Recipient cell	Pathway	Function	Reference
Ovarian CA	Protein	Integrin α6, αv, β1	Epithelial CA cells	N/A	Biomarker	Hurwitz & Meckes Jr (2019)
Ovarian CA	Protein	CD24	Ovarian CA cells	N/A	Biomarker	Soltész *et al.* (2019)
Ovarian CA	Protein	TUBB3, EpCAM, CLDN3, PCNA	Ovarian CA cells	N/A	Biomarker	Liang *et al.* (2013)
Ovarian CA	Protein	COL5A2, LPL	Ovarian CA cells	N/A	Biomarker	Cheng *et al.* (2020)
Ovarian CA	Protein	sHsp	Ovarian CA cells	N/A	Biomarker	Wyciszkiewicz *et al.* (2019)
Ovarian CA	Protein	Plasma gelsolin (pGSN)	Ovarian CA cells	N/A	↑ resistance	Asare-Werehene *et al.* (2020)
Ovarian CA	Protein	DNMT1	Ovarian CA cells	Stimulating endogenous expression	↑ resistance	Cao *et al.* (2017)
Cervical CA	Protein	Hh protein	Cervical CA cells	N/A	Biomarker	Bhat *et al.* (2018)
Ovarian CA	Protein	CLDN4	Ovarian CA cells	Exosome expression	Diagnostic: early detection	Bazzan *et al.* (2021)
Ovarian CA	Protein	TGF-β1	Ovarian CA cells	Tumor progression signaling	Monitoring therapeutic response	Bazzan *et al.* (2021)
Ovarian CA	Protein	MAGE3/6	Ovarian CA cells	Tumor progression signaling	Monitoring therapeutic response	Bazzan *et al.* (2021)
Ovarian CA	miRNA	miR-200b	Ovarian CA cells	Tumor progression signaling	Therapeutic: target for inhibiting tumor progression	Bazzan *et al.* (2021)
Ovarian CA	miRNA	miR-200c	Ovarian CA cells	Tumor progression signaling	Therapeutic: target for inhibiting tumor progression	Bazzan *et al.* (2021)
Ovarian CA	miRNA	miR-21-3p	Ovarian CA cells	Targets NAV3 → apoptosis suppression → chemoresistance	Therapeutic: target for inhibiting chemoresistance	Bazzan *et al.* (2021)
Ovarian CA	miRNA	miR-221-3p	Ovarian CA cells	Inhibiting CDKN1B	↑ progression, ↓ prognosis	Li & Tang (2020)
Ovarian CA	miRNA	miR-141-3p	Endothelial cells	↑ VEGFR-2 expression	↑ migration, angiogenesis	Masoumi-Dehghi *et al.* (2020)
Ovarian CA	miRNA	miR-205	Endothelial cells	PTEN–AKT	↑ angiogenesis, metastasis	He *et al.* (2019*a*)
Ovarian CA	miRNA	miR-21	Ovarian CA cells	Combining with APAF1	↓ apoptosis	Au Yeung *et al.* (2016)
Ovarian CA	miRNA	miR-29a-3p, miR-21-5p	CD4+ T cells	Suppressing STAT3, regulating Treg/Th17	↑ progression, metastasis	Li *et al.* (2014)
Ovarian CA	miRNA	miR-21-3p, miR-21-5p, miR-891-5p	Ovarian CA cells	↑ detoxification and DNA repair	↑ resistance	Alharbi *et al.* (2020)
Ovarian CA	miRNA	miR-223	Hypoxic macrophages	PTEN–PI3K/AKT	↑ resistance	Zhu *et al.* (2019)
Ovarian CA	miRNA	miR-1246	Ovarian CA cells	Targeting Cav1	↑ resistance	Kanlikilicer *et al.* (2018)
Ovarian CA	miRNA	miR-98-5p	Ovarian CA cells	Inhibiting CDKN1A	↑ resistance	Guo *et al.* (2019)
Ovarian CA	miRNA	miR-199a-3p	Ovarian CA cells	Inhibiting C-Met	↓ proliferation, invasion	Kobayashi *et al.* (2020)
Cervical CA	miRNA	Let-7d-3p	Cervical CA cells	Circulating exosome expression	Diagnostic: early detection	Roy *et al.* (2022)
Cervical CA	miRNA	miR-30d-5p	Cervical CA cells	Circulating exosome expression	Diagnostic: early detection	Roy *et al.* (2022)
Cervical CA	miRNA	miR-221-3p	Endothelial cells	Regulating MAPK10	↑ proliferation, invasion, angiogenesis	Zhang *et al.* (2019*a*)
Cervical CA	miRNA	miR-155-5p	Cervical CA cells	↑ IL-6, IL-8	↑ malignancy	Li *et al.* (2019*b*)
Endometrial CA	miRNA	miR-133a	Endometrial CA cells	Downregulating FOXL2	↑ progression	Shi *et al.* (2020)
Endometrial CA	miRNA	miR-148b	Endometrial CA cells	Targeting DNMT1	↓ metastasis	Li *et al.* (2019*a*)
Endometrial CA	miRNA	miR-320a	Endometrial CA cells	Downregulating HIF1 → ↓ VEGFA	↓ proliferation	Zhang *et al.* (2020)
Endometrial CA	miRNA	miR-148b	CAFs	Growth inhibition	Therapeutic: target for inhibition	Bai *et al.* (2020)
Ovarian CA	lncRNA	aHIF	Ovarian CA cells	N/A	Biomarker	Tang *et al.* (2019)
Ovarian CA	lncRNA	MALAT1	HUVECs	VEGF-A, VEGF-D, angiogenin, etc.	Inhibits caspase-3; apoptosis regulation	Qiu *et al.* (2018)
Ovarian CA	lncRNA	UCA1	Ovarian CA cells	Regulates miR-143/FOSL2	↑ resistance	Li *et al.* (2019*d*)
Ovarian CA	circRNA	circPUM1	Mesothelial cells	NF-κB, MMP2 via miR-615-5p/miR-6753-5p	↑ proliferation, migration, invasion	Guan *et al.* (2019)
Ovarian CA	DNA	Wb-mtDNA	Ovarian CA cells	N/A	↑ progression	Keserű *et al.* (2019)
Cervical CA	lncRNA	EXOC7	Cervical CA cells	N/A	Diagnostic biomarker	Guo *et al.* (2020)
Cervical CA	lncRNA	HOTAIR, MALAT1, MEG3	Cervical CA cells	N/A	Biomarker	Zhang *et al.* (2016)
Cervical CA	lncRNA	TUG1	Cervical CA cells	N/A	↑ proliferation, ↓ apoptosis	Hu *et al.* (2017)
Cervical CA	lncRNA	TUG1	Cervical CA cells	Caspase-3, apoptosis proteins	↑ angiogenesis	Lei & Mou (2020)
Cervical CA	circRNA	Hsa-circRNA-0005795, hsa-circRNA-0088088	Cervical CA cells	N/A	Diagnostic, therapeutic	Wang *et al.* (2019*a*)
Cervical CA	circRNA	circEIF4G2	Cervical CA cells	N/A	Biomarker	Mao *et al.* (2020)
Cervical CA	DNA	ATF1, RAS	Cervical CA cells	N/A	Biomarker	Shi *et al.* (2017)

CA, cancer; OC, ovarian cancer; EC, endometrial cancer; CC, cervical cancer; EpCAM, epithelial cell adhesion molecule; CLDN3, claudin 3; PCNA, proliferating cell nuclear antigen; COL5A2, collagen type V alpha 2 chain; LPL, lipoprotein lipase; sHsp, small heat shock protein; pGSN, plasma gelsolin; DNMT1, DNA (cytosine-5)-methyltransferase 1; Hh, hedgehog protein; miRNA/miR, MicroRNA; lncRNA, long noncoding RNA; circRNA, circular RNA; DNA, deoxyribonucleic acid; MALAT1, metastasis-associated lung adenocarcinoma transcript 1; UCA1, urothelial carcinoma-associated 1; NF-κB, nuclear factor kappa-light-chain-enhancer of activated B cells; MMP2, matrix metalloproteinase 2; CDKN1B, cyclin-dependent kinase inhibitor 1B; CDKN1A, cyclin-dependent kinase inhibitor 1A; PTEN, phosphatase and tensin homolog; AKT, protein kinase B; MAPK10, mitogen-activated protein kinase 10; THBS2, thrombospondin 2; STAT3, signal transducer and activator of transcription 3; Treg, regulatory T cell; VEGF, vascular endothelial growth factor; VEGFA, vascular endothelial growth factor A; VEGFR-2, vascular endothelial growth factor receptor 2; PlGF, placental growth factor; ENA-78, epithelial neutrophil-activating peptide 78 (CXCL5); bFGF, basic fibroblast growth factor; APAF1, apoptotic protease-activating factor 1; wb-mtDNA, whole blood–mitochondrial DNA; HIF1α, hypoxia-inducible factor 1 alpha; RAS, rat sarcoma viral oncogene homolog; ATF1, activating transcription factor 1; N/A, not applicable.

### Exosomes in female fertility preservation

A growing body of evidence now supports the use of stem cell-derived exosomes as effective tools for preserving female fertility and promoting reproductive recovery. Importantly, these nanoscale vesicles act as cell-free mediators capable of restoring ovarian and uterine function and improving fertility-related outcomes (Chiu *et al.* 2018). In preclinical models relevant to fertility preservation, MSC-derived exosomes (MSC-Exos) have consistently been shown to enhance follicle survival and support endocrine recovery by improving granulosa cell viability and limiting apoptosis (Borghese *et al.* 2020). Consequently, these protective effects are often accompanied by restoration of ovarian reserve markers and improved follicular architecture, reinforcing the concept that exosomes function as regenerative signals within the ovary (Fig. 4).

In addition to direct cellular protection, angiogenesis plays a pivotal role in successful ovarian recovery, and exosomes have been repeatedly linked to vascular support. For instance, adipose-derived MSC exosomes improve endothelial cell function and stimulate angiogenic signaling, thereby helping to reestablish the microvascular environment required for folliculogenesis and corpus luteum formation (Casagrande *et al.* 2019). At the same time, exosome-mediated anti-inflammatory activity further contributes to ovarian preservation. By suppressing NF-κB activation and reducing pro-inflammatory cytokine expression, exosomes create a more permissive ovarian milieu that supports follicle maintenance and growth (Chu *et al.* 2019).

Beyond ovarian protection, therapeutic exosomes have also demonstrated promising regenerative effects within the uterus. In models of thin endometrium, umbilical cord MSC-derived exosomes improved endometrial epithelial cell viability while attenuating inflammatory signaling (Liang *et al.* 2021). Similarly, in Asherman’s syndrome, adipose-derived MSC exosomes promoted tissue remodeling, enhanced angiogenesis, and facilitated regeneration of a more functional endometrial structure (Gharibeh *et al.* 2022). Taken together, these findings highlight a coordinated role for MSC-derived exosomes in supporting both ovarian and endometrial recovery through integrated effects on cell survival, vascularization, and inflammatory balance.

Finally, pharmacological strategies aimed at fertility preservation are continuing to expand. Tamoxifen has been reported to exert ovarian protective effects in gonadotoxic settings (Nynca *et al.* 2023). Notably, transcriptomic analyses suggest that lncRNA-mediated regulation contributes to these protective mechanisms in tumor-bearing models (Oktay *et al.* 2003, Swigonska *et al.* 2024). Within the context of oncofertility, these observations provide a strong rationale for further investigating exosome-associated non-coding RNAs as therapeutic mediators or biomarkers of female fertility preservation following cancer treatment.

### Exosomes in male fertility preservation

In male oncofertility, therapeutic strategies increasingly focus on restoring spermatogenesis, protecting germ cells, and re-establishing endocrine function. Alongside established fertility preservation approaches – such as sperm cryopreservation and testicular tissue cryopreservation (Goossens *et al.* 2020) – exosome-based therapy is emerging as a promising cell-free modality with the potential to support testicular repair and functional recovery (Vickram *et al.* 2021).

In particular, MSC-derived exosomes have demonstrated notable regenerative effects in preclinical models. These vesicles promote germ cell survival and enhance spermatogenic activity by activating pro-survival and stress-response signaling pathways. For example, bone marrow MSC-derived exosomes protected spermatogonia and supported recovery of spermatogenesis in gonadotoxic injury models (Guo *et al.* 2021). Moreover, related studies indicate that MSC-derived exosomes can facilitate hormonal recovery and improve overall testicular function, including restoration of testosterone production in chemotherapy-associated hypogonadism models (Liang *et al.* 2023).

Beyond stem cell sources, testicular cell-derived exosomes provide an additional and physiologically relevant therapeutic avenue (Gao *et al.* 2023). Sertoli cell-derived exosomes carry microRNAs that regulate apoptosis, proliferation, and differentiation of SSCs, underscoring their role as endogenous regulators of spermatogenic recovery (Wang *et al.* 2023). A2t the same time, exosome-mediated communication between Sertoli and Leydig cells contributes to the regulation of steroidogenesis and maintenance of testicular homeostasis, thereby supporting recovery of endocrine function (Ma *et al.* 2021).

In addition, alternative regenerative exosome sources are being actively explored. Amniotic fluid-derived exosomes have been shown to improve spermatogenesis indices and sperm parameters in azoospermia models, suggesting that they may represent an accessible and effective cell-free option for future translational applications (Mobarak *et al.* 2021). Taken together, these findings position exosomes as promising therapeutic candidates for male fertility preservation, with mechanistic relevance to germ cell support, intercellular communication within the testis, and recovery of endocrine function following gonadotoxic cancer therapies.

### Exosomes as delivery platforms and nanotherapeutics in oncofertility

**Figure 5:**
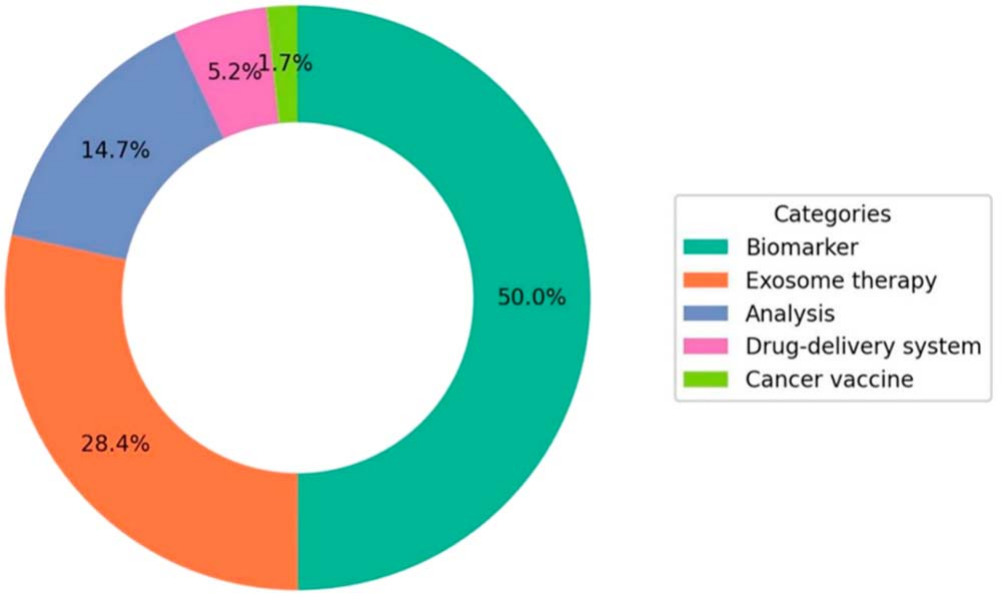
Distribution of exosome applications, showing biomarkers, exosome therapy, analysis, drug delivery systems, and cancer vaccines.

Beyond their regenerative roles, exosomes are increasingly recognized as versatile delivery platforms that may support fertility-sparing cancer therapies in oncofertility settings (Fig. 5). Owing to their nanoscale size, intrinsic stability, low immunogenicity, and ability to cross biological barriers, exosomes provide a natural nanocarrier system capable of improving therapeutic precision while potentially limiting off-target toxicity to reproductive tissues (Chen *et al.* 2021*a*). Nevertheless, challenges such as rapid systemic clearance and limited tissue targeting remain barriers to clinical translation. To address these limitations, bioengineering strategies have been developed, including internal cargo loading with drugs, nucleic acids, or gene-editing tools, as well as surface modification to enhance tissue-specific delivery (Patil *et al.* 2020, Jahanbani *et al.* 2023*a*).

In gynecological cancers, exosome-based nanotherapeutic approaches have demonstrated promising anticancer efficacy with improved targeting profiles. In ovarian cancer, exosomes loaded with miR-199a-3p suppressed C-met signaling, resulting in reduced tumor cell proliferation and aggressiveness (Yumura *et al.* 2018). More advanced strategies include tumor-derived exosomes engineered to deliver CRISPR/Cas9 plasmids, which enabled efficient silencing of PARP-1 and induction of apoptosis in ovarian cancer cells (Kim *et al.* 2019). In endometrial cancer, cancer-associated fibroblast-derived exosomes carrying miR-320a inhibited tumor growth by downregulating HIF-1α and VEGFA expression, further highlighting the versatility of exosome-mediated delivery systems (Zhang *et al.* 2020).

Additional studies underscore the adaptability of exosome-based nanoplatforms for drug and nucleic acid delivery. Exosome membrane-coated nanoparticles improved siRNA stability and enhanced *in vivo* tumor suppression (Zhao *et al.* 2020), while aspirin-loaded exosomes increased drug solubility and cytotoxic efficacy (Tran *et al.* 2019). In cervical cancer models, MSC-derived exosomes delivering inhibitory oligonucleotides targeting miR-142-3p and miR-150 significantly reduced tumor progression (Naseri *et al.* 2018). Moreover, milk-derived exosomes transporting anthocyanidins or curcumin demonstrated superior antiproliferative and anti-inflammatory effects compared with free compounds in ovarian and cervical cancer models (Aqil *et al.* 2017, Bai *et al.* 2020).

Furthermore, preclinical studies demonstrate that exosome-based and synthetic nanocarrier systems can efficiently deliver small interfering RNAs (siRNAs) and other nucleic acid cargos to tumor cells, achieving robust gene silencing while limiting off-target exposure (Jangde *et al.* 2025). In prostate cancer (PC) models, lipid nanoparticles, polymeric carriers, metallic nanoparticles, and exosome-mimetic vesicles have been shown to enhance siRNA stability, intracellular uptake, and tumor selectivity (Saeinasab *et al.* 2024).

Targeted delivery of oncogene-directed siRNAs (e.g., EGFR, AR, and SNHG15) using ligand-functionalized nanocarriers – such as PSMA-, folate-, or EpCAM-targeted systems – resulted in marked tumor growth inhibition, apoptosis induction, and suppression of metastatic potential *in vitro* and in xenograft models (Heris *et al.* 2023, Saeinasab *et al.* 2024). Importantly, by improving delivery precision, these approaches may reduce dependence on aggressive chemoradiotherapy, thereby indirectly preserving spermatogenesis and endocrine balance (Jangde *et al.* 2025).

From a reproductive safety perspective, preclinical evidence also highlights potential risks. Nanocarriers may accumulate in non-tumor tissues, including testes, depending on size, surface charge, and biodegradability (Jangde *et al.* 2025). Although siRNA sequence specificity limits unintended gene silencing, experimental models indicate that poorly optimized nanocarriers can impair spermatogenesis, alter testicular architecture, and disrupt hormonal signaling (Shafi *et al.* 2013). These findings emphasize the need for fertility-aware nanocarrier design and biodistribution profiling.

Recent preclinical work has expanded the scope of exosome-based fertility preservation by exploring *cell*-free, primed sEVs as nanotherapeutics for premature ovarian insufficiency (POI) (Seegobin *et al.* 2025). In a cyclophosphamide-induced granulosa cell (GC) injury model, primed avian MSC-derived sEVs (primed AMSC-sEVs) demonstrated superior physicochemical stability and bioactivity compared with naïve sEVs, exhibiting smaller and more uniform particle size, higher vesicle concentration, and increased negative zeta potential – features associated with enhanced cellular uptake and therapeutic performance (Shieh *et al.* 2025). Functionally, primed sEVs restored GC proliferation, suppressed apoptosis, and rescued steroidogenic capacity, reversing chemotherapy-induced reductions in AMH, FSHR, LHCGR, estradiol, and progesterone. These effects were accompanied by downregulation of pro-apoptotic markers (cleaved caspase-3, BAX, and PARP) and upregulation of BCL-2. Mechanistically, next-generation sequencing revealed enrichment of fertility- and survival-associated miRNAs, including miR-21, miR-22, miR-23b, miR-145, and miR-199a, implicating coordinated post-transcriptional regulation of apoptosis and steroidogenesis. Although confined to *in vitro* human granulosa cell models, this study provides compelling proof-of-concept evidence that engineered or primed sEVs can enhance the regenerative efficacy of exosome-based nanotherapies, supporting their future translation for fertility preservation and early ovarian insufficiency management (Shieh *et al.* 2025).

Beyond direct drug delivery, engineered exosomes also offer opportunities for immunomodulation and biodistribution control. For example, exosomes have been engineered to modulate immune responses, redirecting effector cell activity and opening new avenues for cancer immunotherapy (Cheng *et al.* 2018). In parallel, modulation of nanoparticle biodistribution using peripheral blood-derived exosomes has been shown to reduce hepatic accumulation and enhance therapeutic efficiency of plant-derived nanovectors (Wang *et al.* 2018).

On the other hand, tumor-derived exosomes actively shape anti-tumor immunity by transferring immunosuppressive cargos, including immune checkpoint molecules, cytokines, and regulatory RNAs, thereby modulating T-cell activation and antigen presentation (Caserta *et al.* 2024). Notably, exosomal PD-L1 has been shown to suppress cytotoxic T-cell responses and contribute to resistance against immune checkpoint inhibitors, positioning exosomes as both biomarkers and functional mediators of immunotherapy response (Silva *et al.* 2019). Conversely, immune cell-derived and engineered exosomes are being explored as novel immunotherapeutic platforms capable of delivering tumor antigens, immune-stimulatory RNAs, or drugs to enhance anti-tumor immunity with reduced systemic toxicity (Huang *et al.* 2023).

Preclinical studies have also highlighted the regenerative potential of umbilical cord-derived MSC exosomes (UC-MSC-Exos) as a cell-free alternative for treating intrauterine adhesions (IUAs) (Xin *et al.* 2022). In a rat model of endometrial injury, UC-MSC-Exos incorporated into a collagen scaffold (CS/Exos) significantly enhanced endometrial regeneration, promoted collagen remodeling, restored estrogen receptor-α and progesterone receptor expression, and ultimately recovered fertility (Xin *et al.* 2020). Mechanistically, CS/Exos therapy exerted strong immunomodulatory effects, driving CD163^+^ M2 macrophage polarization, suppressing local inflammation, and enhancing anti-inflammatory signaling both *in vivo* and *in vitro*. RNA-sequencing analyses identified exosome-enriched miRNAs as key mediators of macrophage reprogramming and tissue repair. Although limited to animal models, this work provides compelling proof-of-concept that combining exosomes with biomaterial scaffolds can overcome poor retention and delivery efficiency, supporting their future translation as regenerative therapies. Although this study was not conducted in an oncological setting, the regenerative mechanisms identified are highly relevant to fertility preservation and may inform future oncofertility interventions aimed at restoring uterine function after cancer treatment (Xin *et al.* 2020).

In ovarian cancer models, tumor-derived exosomes promote immune suppression by enhancing regulatory T-cell (Treg) activity and inhibiting cytotoxic immune responses through immunosuppressive factors such as TGF-β1 and interleukin-10 (IL-10) (Gong *et al.* 2023). Exosomes isolated from ovarian cancer ascites have been shown to induce apoptosis of peripheral blood lymphocytes and dendritic cells (DCs), further impairing anti-tumor immune surveillance (Inagaki *et al.* 2016, Nakamura *et al.* 2019). At the same time, ovarian cancer-derived exosomes carry tumor-associated antigens, including EpCAM and CD44, supporting their potential use as immunogenic platforms for targeted immunotherapy (Grasso *et al.* 2015). Experimental studies also highlight the complexity of exosome-mediated immune regulation. While dendritic cell-derived exosomes can stimulate T-cell activation, tumor-derived exosomes expressing Fas ligand (FasL), TRAIL, or galectin-9 can induce apoptosis of CD8^+^ T cells and suppress T-cell receptor signaling pathways, thereby promoting immune tolerance (Klibi *et al.* 2009).

Within the context of oncofertility, these delivery-based strategies are particularly relevant, as targeted exosome-mediated therapies may achieve effective tumor control while reducing systemic exposure and preserving reproductive potential. When integrated with regenerative approaches, exosome-based nanotherapeutics represent a complementary strategy aimed at balancing cancer treatment efficacy with long-term fertility preservation in reproductive-age patients.

### Translational and clinical perspectives of exosomes in reproductive disorders

Building on the strong body of preclinical evidence, recent years have witnessed the first steps toward clinical translation of exosome-based regenerative strategies in reproductive medicine. While most data supporting the protective and reparative effects of stem cell-derived exosomes originate from animal models and *in vitro* studies, these findings have laid the groundwork for early-phase human investigations. Accordingly, a limited but growing number of registered clinical trials have now begun to evaluate the safety, feasibility, and preliminary efficacy of exosome-based interventions in patients with reproductive dysfunction. In addition, while clinical translation of exosome-based therapies has begun to emerge in female reproductive disorders corresponding clinical studies in male infertility remain scarce. This disparity highlights a clear translational gap and underscores the need for well-designed clinical trials to evaluate the safety, efficacy, and long-term outcomes of exosome-based therapies in male oncofertility. These studies represent critical milestones in bridging experimental discoveries with real-world clinical applications in oncofertility and fertility preservation (Park *et al.* 2024).

A registered human clinical study (ClinicalTrials.gov Identifier: NCT06841328) is currently investigating the safety and feasibility of intra-gonadal administration of adipose-derived stem cells (ADSCs) or stem cell-derived exosomes in patients with gonadal failure, including POF, ovarian insufficiency, hypogonadism, and testicular dysfunction. This open-label, single-arm pilot study enrolls both male and female participants who have shown inadequate responses to conventional treatments such as hormone replacement therapy or assisted reproductive technologies. The intervention involves direct intraovarian or intratesticular injection, with longitudinal follow-up at 3, 6, 9, and 12 months to assess hormonal recovery (e.g. estradiol, testosterone, follicle stimulating hormone (FSH), and AMH), structural gonadal changes, reproductive function, and treatment-related adverse events.

Beyond gonadal regeneration, clinical translation of exosome-based therapies has also expanded toward uterine and endometrial repair, addressing another major cause of infertility in cancer survivors and women with refractory reproductive disorders. In contrast to intra-gonadal approaches, recent clinical efforts have focused on the regenerative capacity of stem cell-derived exosomes within the endometrium, particularly in cases of thin endometrium and severe intrauterine adhesions. In this context, a registered prospective clinical study (ClinicalTrials.gov ID: NCT06896747) is currently evaluating the safety and efficacy of umbilical cord-derived MSC exosomes for the treatment of thin endometrium secondary to severe intrauterine adhesions. This non-randomized, parallel-controlled trial compares mechanically engineered exosomes with conventional exosomes and platelet-rich plasma (PRP), a widely used regenerative intervention in reproductive medicine. Participants receive a single hysteroscopically guided subendometrial injection during the proliferative phase of the menstrual cycle. The primary endpoint is improvement in endometrial thickness, while secondary outcomes include implantation rate, clinical pregnancy rate, live birth rate, miscarriage rate, and safety assessments. Importantly, this study introduces mechanically engineered exosomes, produced through biophysical modulation during stem cell culture, with the aim of enhancing regenerative potency compared to conventional exosomes.

Moreover, Reza *et al.* (2016) demonstrated that conditioned medium derived from human adipose mesenchymal stem cells (hAMSCs) suppressed ovarian cancer (OC) cell growth by inducing cell cycle arrest and mitochondria-mediated apoptosis. Notably, exosomes isolated from hAMSC-conditioned medium enhanced these effects through upregulation of pro-apoptotic markers, including BAX, CASP9, and CASP3, and downregulation of the anti-apoptotic protein BCL2. Exosomal miRNAs were identified as key mediators of these anticancer effects (Reza *et al.* 2016).

In parallel, Li *et al.* reported that exosomes isolated from malignant ascites of OC patients carried tumor-associated antigens capable of being presented by dendritic cells derived from unrelated cord blood, leading to tumor-specific cytotoxic immune responses. This finding supports the use of exosome-based immunotherapeutic strategies in OC (Li *et al.* 2008*a*). Furthermore, Bretz *et al.* showed that ascites-derived exosomes activated Toll-like receptor-dependent pathways in mononuclear precursor cells, triggering broader immune activation. These observations underscore the role of exosomes in shaping tumor immunity.

Investigation has explored the regenerative potential of autologous platelet-derived exosomes for ovarian rejuvenation in women with diminished ovarian reserve. The Exosomas 2024-1 trial (ClinicalTrials.gov ID: NCT06773572) represents one of the first randomized, double-blind clinical studies directly comparing exosome-based ovarian therapy with platelet-derived growth factors and placebo. This prospective pilot study enrolled 30 women aged 38–46 years with established diminished ovarian reserve who declined oocyte donation. Participants were randomized into three treatment arms receiving intraovarian injections of i) autologous platelet-derived exosomes, ii) activated platelet growth factors (PRP), or iii) physiological saline as control. Treatments were administered monthly over four consecutive menstrual cycles during the early follicular phase. The rationale for this intervention was grounded in age-related ovarian stromal fibrosis, mitochondrial dysfunction, oxidative stress, and depletion of key regenerative signals, including reduced expression of exosomal markers (CD63 and CD81) and regulatory microRNAs (miR-21, miR-125, miR-132, and miR-199). Autologous exosomes were isolated from platelet-rich plasma using standardized protocols, yielding highly concentrated vesicle preparations (approximately 5–6 trillion exosomes/mL) for targeted ovarian delivery.

Clinical outcomes were evaluated through hormonal profiling (FSH, estradiol, and AMH) and antral follicle count, measured before treatment initiation and after the final intervention. By directly comparing exosomes with PRP and placebo, this study provides early clinical evidence suggesting that cell-free exosome therapy may exert superior regenerative effects on ovarian function relative to conventional platelet-based approaches.

Translation of exosome-based immunotherapy into the clinic has been most advanced in the context of dendritic cell-derived exosomes (Dexs). Following the seminal discovery by Raposo *et al.* (1996) that exosomes participate in antigen presentation and acquired immunity, Dexs were developed as cell-free cancer vaccines capable of delivering tumor antigens and major histocompatibility complex (MHC) molecules to elicit antigen-specific T-cell responses (Gong *et al.* 2023). Importantly, phase I clinical trials have confirmed the feasibility and safety of Dex-based vaccines, demonstrating that exosome-based immunotherapies can be administered to patients without significant toxicity while inducing measurable immune activation (Jaiswal & Sedger 2019, Saumell-Esnaola *et al.* 2022). These trials represent a pivotal milestone that enabled the transition of exosome research from experimental immunology to clinical oncology.

In ovarian cancer, translational studies further support immunotherapeutic relevance, as higher intratumoral T-cell infiltration correlates with improved survival outcomes (Yang *et al.* 2020*b*). Building on these observations, combination strategies integrating exosome-based antigen delivery with immune adjuvants, such as Toll-like receptor-3 (TLR3) agonists, have been proposed to overcome tumor-induced immune tolerance and potentially prolong progression-free survival in patients with high-grade ovarian cancer (Adams *et al.* 2005).

Collectively, these studies demonstrate that exosomes derived from MSCs or tumor-associated fluids can modulate apoptosis, antigen presentation, and immune signaling in ovarian cancer. Within the context of oncofertility, such approaches are particularly relevant, as exosome-based therapies may enable effective tumor control while reducing systemic toxicity and preserving reproductive potential. Accordingly, exosomes represent promising tools for integrating cancer treatment with fertility preservation strategies in female reproductive system malignancies (Table 3).

**Table 3:** Clinical and experimental applications of exosomes in oncofertility and related cancers.

Cancer/disease	Exosome/target	Donor cell/origin	Recipient cell	Pathway/method	Function/goal	Reference
Colorectal cancer	Circulating exosomes	Patient serum	–	Tumor exosome transporter protein assays	Diagnosis: ↑ sensitivity/specificity for CRC	NCT04394572
Gastric cancer	lncRNA-GC1	Peripheral blood	–	Circulating exosomal lncRNA-GC1	Predicting and monitoring immunotherapy outcomes	NCT05334849
Lung cancer	lncRNAs	Plasma	–	RNA sequences	Screening for early lung cancer	NCT03830619
Colon cancer	Plant-derived exosomal curcumin	Plant sources	Colon CA cells	Oral absorption and delivery	Therapeutic: Inhibit progression	NCT01294072
Metastatic pancreatic CA	MSC-exos (KrasG12D siRNA)	MSCs	Pancreatic CA cells	siRNA delivery	Treatment: max tolerated dose and toxicity	NCT03608631
Non-small cell lung CA	DC-exosomes with tumor antigens	Dendritic cells	Lung CA cells	Antigen presentation	Therapy: Improve survival	NCT01159288
Breast cancer	Circulating exosomes	Serum after chemotherapy and surgery	–	Biomarker evaluation	Diagnosis: Recurrence biomarker	NCT05955521
EOC	miR-200b	Plasma	Ovarian CA cells	Exosomal miRNA enrichment	Diagnostic and prognostic biomarkers	Nakayama *et al.* (2013)
Pancreatic cancer	KRAS and p53 EVs	Serum	–	Exosome biomarker analysis	Predictive markers for pancreatic CA	Kahlert *et al.* (2014)
Breast/prostate/other tumors	Various miRNAs/lncRNAs	Plasma/serum	–	Exosome profiling	Prognostic and diagnostic markers	Sakaue *et al.* (2019), Wang *et al.* (2024)
Breast CA	miR-23b exosomes	MSCs	Breast CA cells	Targeting MARCKS	Therapy	Ono *et al.* (2014)
Breast CA	EMs	MSCs	Breast CA cells	Drug delivery	Therapy	Kalimuthu *et al.* (2018)
Breast CA	miR-379 exosomes	MSCs	Breast CA cells	Drug delivery	Therapy	O’Brien *et al.* (2018)
Ovarian CA	EMs	hAMSCs	Ovarian CA cells	Blocking cell cycle + apoptosis	Therapy	Reza *et al.* (2016)
Ovarian CA	EPs	Malignant ascites	Ovarian CA cells	Tumor antigen presentation	Therapy	Li *et al.* (2008*b*)
Ovarian CA	EPs	Malignant ascites	Ovarian CA cells	Knockdown of TLR2/TLR4 → block NFκB/STAT3	Therapy	Bretz *et al.* (2013)

EOC, epithelial ovarian cancer; EMs, exosome mimetics; and EPs, exosomal proteins.

### Biological limits of regeneration in the reproductive system

Despite the rapid expansion of regenerative research in oncofertility, it is essential to clearly distinguish between functional recovery and true biological regeneration within the reproductive system. Unlike highly regenerative tissues such as skin or liver, reproductive organs operate under strict biological constraints that fundamentally limit their capacity for *de novo* germ cell formation (Mäkelä & Hobbs 2019, Martin *et al.* 2019).

In the female reproductive system, the idea of new oocyte formation in adult ovaries remains highly controversial. The prevailing consensus supports the concept that the ovarian reserve is finite and established early in life, with follicle depletion occurring progressively thereafter (Martin *et al.* 2019). To date, no definitive experimental or clinical evidence has demonstrated sustained *de novo* oogenesis in adult mammals, including humans (Saitou 2009). As a result, interventions described as ‘regenerative’ in ovarian biology typically reflect preservation of existing follicles, enhancement of follicular survival, or restoration of stromal and endocrine support, rather than true germ cell neogenesis (Brinster & Zimmermann 1994, De Rooij 2017, Lord & Oatley 2017).

A similar distinction applies to the male reproductive system. While adult testes retain SSCs that support continuous spermatogenesis, recovery after gonadotoxic injury primarily depends on the survival, activation, and niche support of these pre-existing cells (Mäkelä & Hobbs 2019, Kalluri & LeBleu 2020). Importantly, this process represents functional restoration rather than the generation of entirely new germ cell lineages (Mäkelä & Hobbs 2019).

Within these biological boundaries, exosomes have emerged as potent regulators of cell survival, angiogenesis, immune modulation, and microenvironmental repair. Extensive preclinical evidence shows that exosome-based interventions can improve ovarian and testicular function by reducing apoptosis, limiting oxidative stress, enhancing vascularization, and stabilizing germ cell niches (Yang *et al.* 2020*a*, Chen & Wang 2022, Liu *et al.* 2023, Izadpanah *et al.* 2024). However, despite these benefits, no study to date has conclusively shown that exosomes induce true germ cell neogenesis in either female or male reproductive organs.

Notably, the majority of evidence supporting exosome-mediated fertility restoration arises from animal models, where outcomes are commonly assessed through improved hormone levels, follicle counts, spermatogenic indices, or reproductive performance (Turner *et al.* 2021, Rives *et al.* 2022, Dimik *et al.* 2023). While encouraging, these endpoints reflect functional recovery rather than structural regeneration. In humans, clinical evidence remains limited, and there is no proof that exosome-based therapies can generate new oocytes or germ cells (Jahanbani *et al.* 2023*b*, Ghasroldasht *et al.* 2025).

Taken together, exosomes represent promising tools for fertility preservation and tissue repair, but their regenerative capacity must be interpreted within the inherent biological limits of the reproductive system. Recognizing this distinction is critical for precise terminology, realistic clinical expectations, and responsible translation of preclinical findings into oncofertility practice (Sehring *et al.* 2021, Wyns *et al.* 2021).

## Challenges and future directions

Despite the remarkable progress in understanding the diagnostic and therapeutic potential of exosomes, particularly exosomal lncRNAs, significant challenges still limit their clinical application. Issues related to isolation, characterization, and standardization remain unresolved, while biological questions about selective cargo loading, donor–recipient specificity, and exosome heterogeneity continue to complicate their use. Addressing these obstacles is essential for advancing exosome-based strategies from experimental models to effective clinical tools in oncology and reproductive medicine.

Therapeutic application faces its own barriers. While exosomes are being engineered as drug delivery systems, limitations include efficient drug loading, optimizing producer–target cell pairing, and ensuring ligand–receptor specificity for targeted uptake. Rapid clearance by macrophages and their short half-life *in vivo* (Parada *et al.* 2021) further reduce efficacy. To counter this, bioengineering approaches, such as combining exosomes with biomaterials, are being explored to improve retention and controlled release at target sites (Chen *et al.* 2022, Jahanbani *et al.* 2022).

Recent studies have highlighted the promise of exosome–biomaterial hybrids. For example, UC-MSC-derived exosomes combined with collagen scaffolds enhanced endometrial repair in rats, not only improving ERα and PR expression but also significantly increasing pregnancy rates (Xin *et al.* 2020). Similarly, PEG hydrogel systems carrying AMSC-derived exosomes provided antibacterial protection and long-term release for endometrial regeneration and fertility restoration (Lin *et al.* 2021).

Exosomal lncRNAs have drawn considerable interest as potential diagnostic and prognostic biomarkers, owing to their secretion by nearly all cell types and detectability in body fluids. However, their clinical translation remains hampered by technical and biological challenges (Hosseini *et al.* 2022).

Isolation and purification remain among the greatest hurdles. Ultracentrifugation yields high-purity vesicles but is time-consuming, costly, and requires specialized equipment, while other methods often result in contamination from lipoproteins or ribonucleoprotein complexes, complicating downstream analysis – especially when RNA yields are low (Hosseini *et al.* 2022). Likewise, RNA extraction methods are highly variable; Eldh *et al.* (2012) demonstrated that yield, purity, and size distribution differ markedly across protocols, emphasizing the need to match methods to research goals (Tellez-Gabriel & Heymann 2019).

Characterization and quantification pose additional limitations. Distinguishing tumor-derived exosomes from those secreted by normal cells remains difficult, particularly in plasma samples where platelet-derived RNAs dominate. The available detection platforms – including flow cytometry, ELISA, and nanoparticle tracking analysis – offer valuable insights but are hindered by high costs, contamination risks, and inconsistent reproducibility (Makler & Asghar 2020). For lncRNA profiling, RT-qPCR is widely used due to affordability and practicality, while NGS and microarrays provide comprehensive data but remain resource-intensive. Digital PCR (dPCR) is emerging as a more sensitive option, but requires further validation (Wu *et al.* 2020).

Biological questions also remain unresolved. Why certain lncRNAs are selectively packaged into exosomes, how donor–recipient specificity is maintained, and the degree to which exosomes alter the activity of their own cargo are still poorly understood (Yuan & Huang 2021). Contradictory findings further complicate interpretation – for instance, Yang *et al.* (2019) reported that HOTAIR upregulation enhanced exosome release in hepatocellular carcinoma, while other studies observed the opposite effect (Li *et al.* 2021). Moreover, tumors secrete heterogeneous populations of exosomes, each potentially carrying distinct molecular signatures. Systematically classifying these subtypes – akin to the classification of blood cells – could enhance their precision as biomarkers and therapeutic agents (Karamian *et al.* 2021).

Finally, the field is witnessing a transition from discovery to translation. Early research focused heavily on plasma and serum exosome profiling for biomarker discovery. Today, attention is shifting toward clinical validation, with studies evaluating efficacy, safety, treatment combinations, and measurable outcomes (Mohan *et al. *2026). This evolution underscores the shift from foundational research toward applied clinical applications in oncology and reproductive medicine.

## Conclusion

In conclusion, chemotherapy effectively treats cancer but often causes long-term harm to reproductive function in both women and men. These effects represent a major quality-of-life issue for cancer survivors. Exosomes have emerged as important players in this process, reflecting cellular damage while also offering new opportunities for diagnosis and therapy. Their ability to carry biologically active molecules makes them valuable biomarkers of reproductive toxicity and promising tools for fertility protection and restoration. Although technical and biological challenges still limit their clinical use, continued research, improved standardization, and careful clinical evaluation may enable exosomes to bridge cancer treatment and reproductive health. Ultimately, they hold the potential to support cancer survival while preserving reproductive capacity.

## Declaration of interest

The authors have no relevant financial or non-financial interests to disclose.

## Funding

This work did not receive any specific grant from any funding agency in the public, commercial, or not-for-profit sector.

## Author contribution

MMA and IMS conceived the study. MMA, BA, KA, AA, and IMS prepared the original draft of the manuscript. MMA, BA, KA, AA, and IMS reviewed and edited the manuscript. All authors have read and agreed to the published version of the manuscript.
